# Supplementary figures and images for: The PARP Inhibitor Olaparib Modulates the Transcriptional Regulatory Networks of Long Non-Coding RNAs during Vasculogenic Mimicry
Source: Cells. 2020 Dec 15;9(12):2690. doi: 10.3390/cells9122690 (PMC7765283; doi:10.3390/cells9122690)

Figure S1

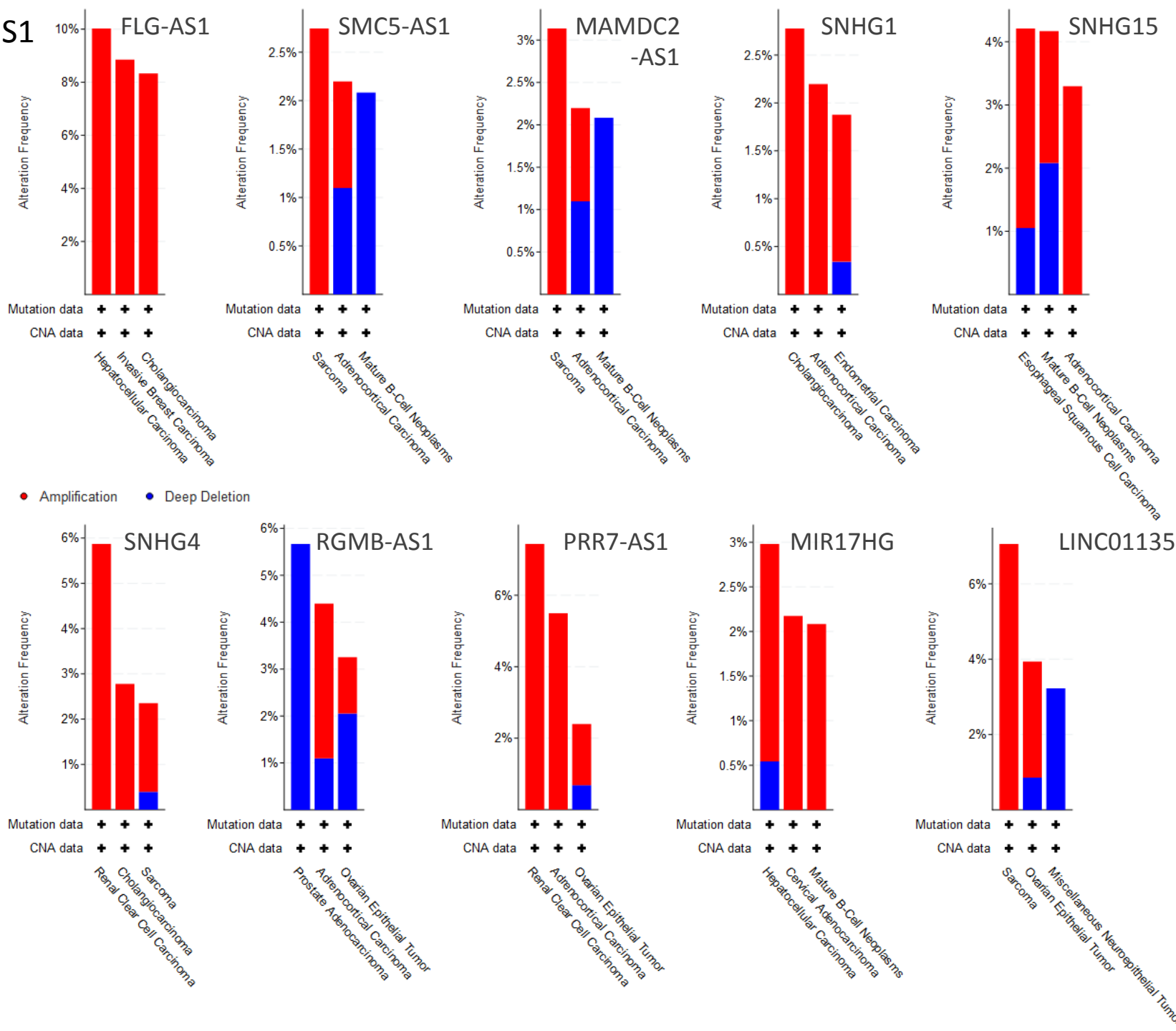

Fig S2

A

SNHG1 vs. PARP2

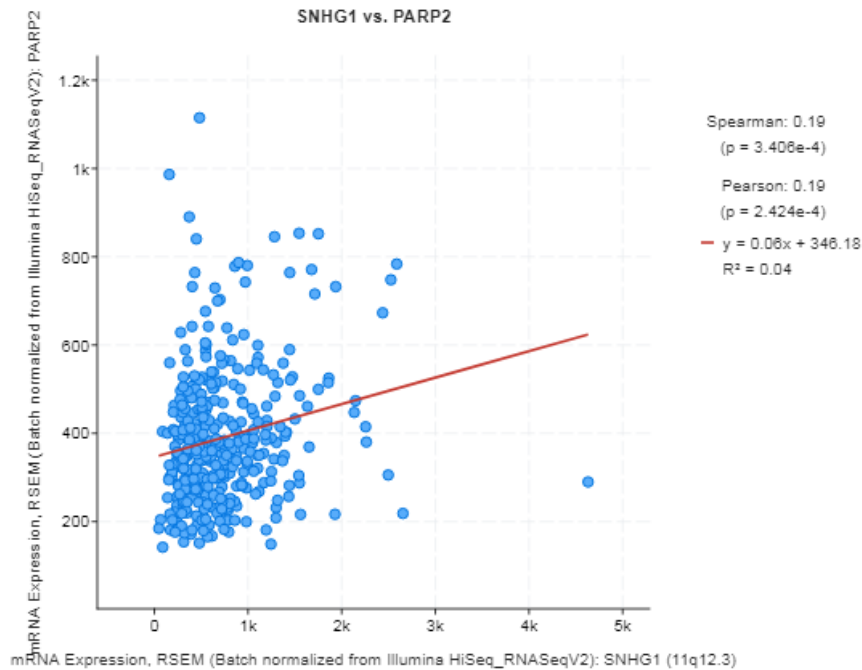

B

SNHG4 vs. PARP1

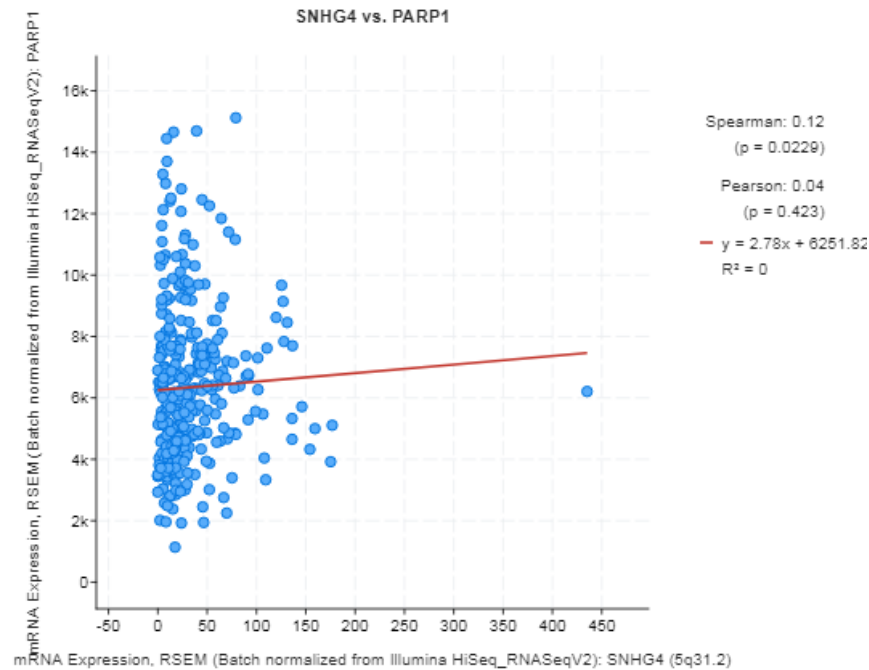

Figure S3

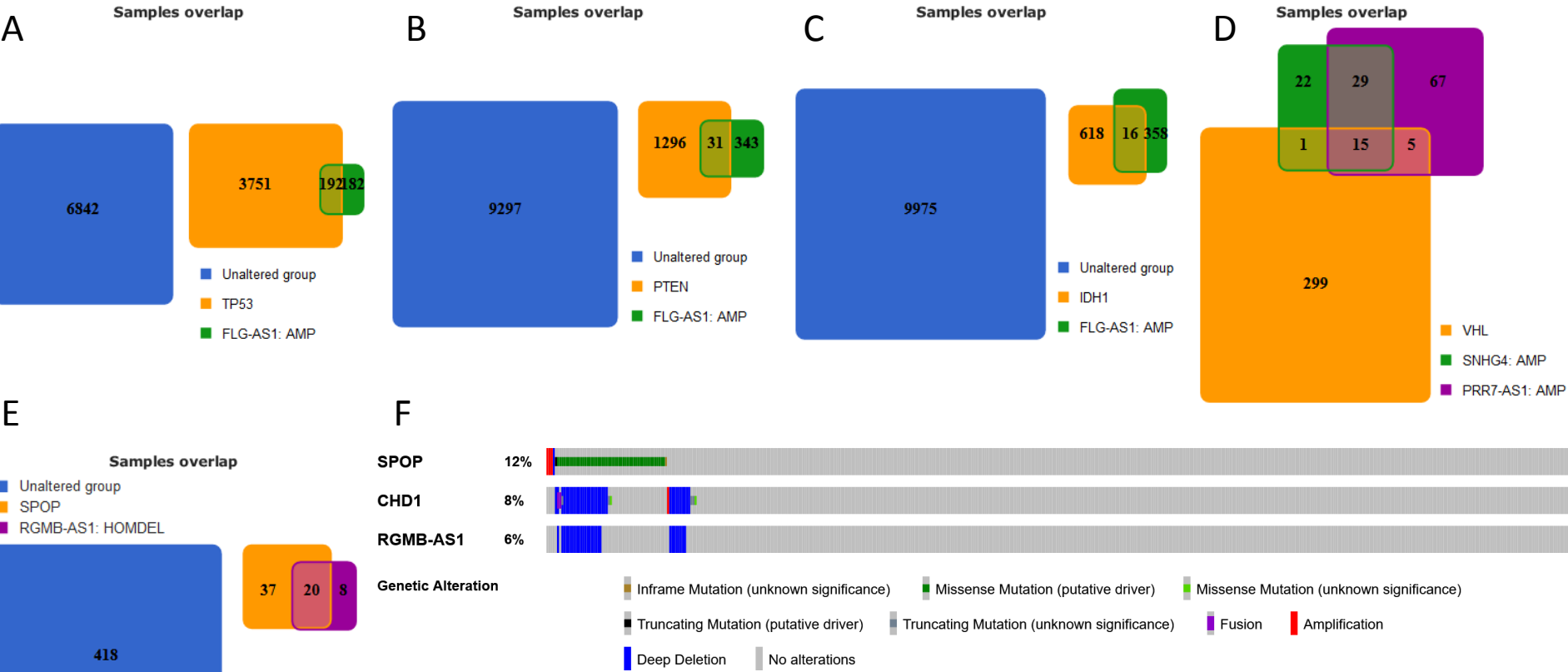

Figure S4

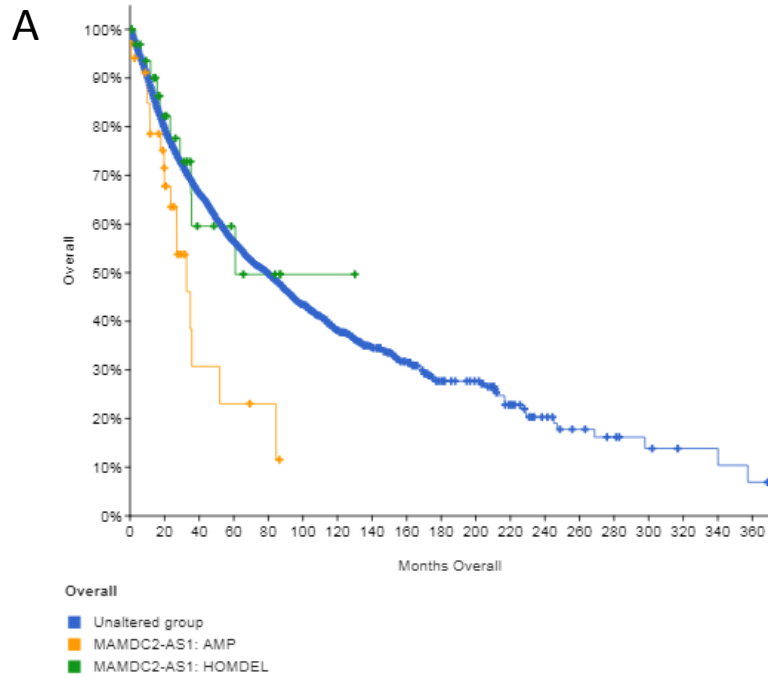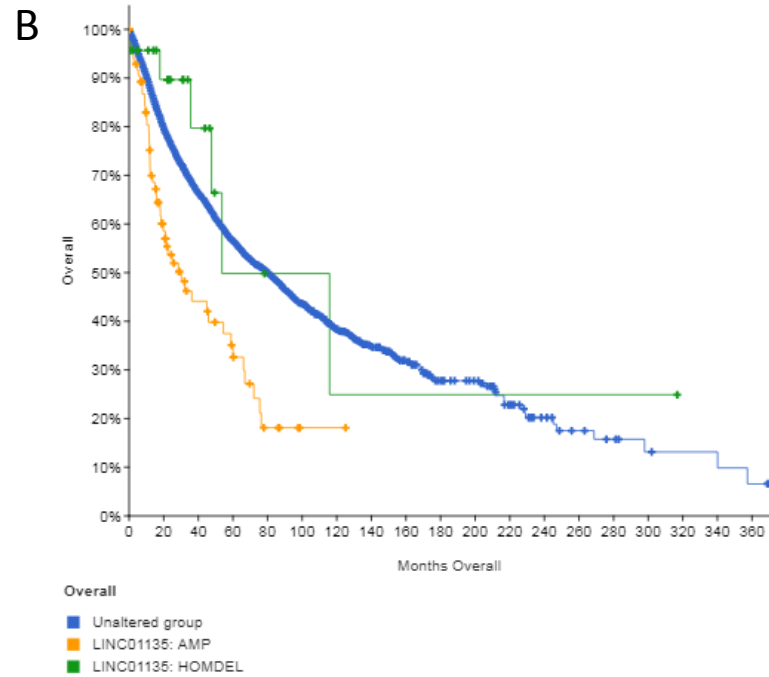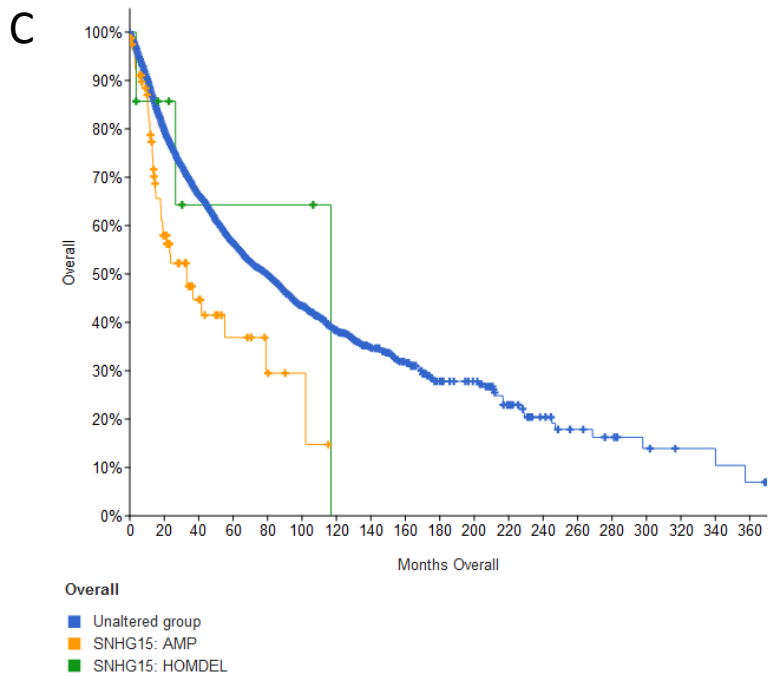

Supplement: Supplementary file 1 [file cells-09-02690-s001.pdf]
